# Bioactive Peptides from *Ruditapes philippinarum* Attenuate Hypertension and Cardiorenal Damage in Deoxycorticosterone Acetate–Salt Hypertensive Rats

**DOI:** 10.3390/molecules28227610

**Published:** 2023-11-15

**Authors:** Zonghui Sun, Weixia Wang, Jinli Liu, Shengcan Zou, Dongli Yin, Chenghan Lyu, Jia Yu, Yuxi Wei

**Affiliations:** 1 College of Life Sciences, Qingdao University, Qingdao 266071, China; szh1840@163.com; 2 Qingdao Chenlan Pharmaceutical Co., Ltd., Qingdao 266061, China; ytqxwwx1990@163.com (W.W.); liu_jinli_ok@126.com (J.L.); zoushengcan@chenland.cn (S.Z.); dongli215@163.com (D.Y.); chenghanlyu@126.com (C.L.)

**Keywords:** *Ruditapes philippinarum* peptides, DOCA–salt hypertensive rats, antihypertension, cardiorenal protection, oxidative stress, gut microbiota

## Abstract

Hypertension is a common disease that affects human health and can lead to damage to the heart, kidneys, and other important organs. In this study, we investigated the regulatory effects of bioactive peptides derived from *Ruditapes philippinarum* (RPP) on hypertension and organ protection in deoxycorticosterone acetate (DOCA)–salt hypertensive rats. We found that RPPs exhibited significant blood pressure-lowering properties. Furthermore, the results showed that RPPs positively influenced vascular remodeling and effectively maintained a balanced water–sodium equilibrium. Meanwhile, RPPs demonstrated anti-inflammatory potential by reducing the serum levels of inflammatory cytokines (TNF-α, IL-2, and IL-6). Moreover, we observed the strong antioxidant activity of RPPs, which played a critical role in reducing oxidative stress and alleviating hypertension-induced damage to the aorta, heart, and kidneys. Additionally, our study explored the regulatory effects of RPPs on the gut microbiota, suggesting a possible correlation between their antihypertensive effects and the modulation of gut microbiota. Our previous studies have demonstrated that RPPs can significantly reduce blood pressure in SHR rats. This suggests that RPPs can significantly improve both essential hypertension and DOAC–salt-induced secondary hypertension and can ameliorate cardiorenal damage caused by hypertension. These findings further support the possibility of RPPs as an active ingredient in functional anti-hypertensive foods.

## 1. Introduction

Hypertension presents a growing global public health concern, including essential and secondary forms triggered by various factors [1]. It is worth noting that approximately 50% of hypertensive patients exhibit salt sensitivity, wherein a high-salt environment escalates oxidative stress levels and triggers an augmented inflammatory response [2]. Prolonged consumption of high-salt diets not only elevates blood pressure but also results in independent side effects leading to target organ damage, particularly the kidneys [3]. The consequences of hypertension-induced target organ damage manifest as cardiac remodeling, cardiac hypertrophy, glomerulosclerosis, tubular atrophy, and interstitial fibrosis, ultimately culminating in irreversible harm to vital organs [4]. Therefore, an ideal antihypertensive therapy should address blood pressure and target oxidative stress to mitigate damage to these critical organs. While there are many antihypertensive drugs available, single-agent therapy often proves less effective, necessitating combination therapy for effective blood pressure management [5]. In addition, the use of antihypertensive drugs in therapy is associated with mild (hyperkalemia, hypotension) and severe adverse events (acute kidney injury, syncope) [6]. As a promising alternative, numerous bioactive peptides have exhibited antihypertensive effects with fewer side effects compared with traditional drugs [7,8,9].

*Ruditapes philippinarum*, commonly known as clams, is widely distributed around the world and holds high economic value. It is one of the most exploited shellfish in the world, with an annual production of up to 4 million tons [10]. The average contents of moisture, protein, ash, fat, and glycogen in *Ruditapes philippinarum* are 77.4%, 10.9%, 3.2%, 1.6%, and 2.8%, respectively [11]. Therefore, it is an ideal raw material for the development of bioactive peptides. Various peptides with notable biological activities have been identified and characterized from clams using diverse preparation methods. For instance, Yang et al. identified and characterized a peptide from the manila clam that exhibits exceptional antibacterial activity [12]. Song et al. obtained bioactive peptides with lipid-lowering activity from *Ruditapes philippinarum* through fermentation via natto bacteria [13]. Additionally, Gao et al. demonstrated that peptides obtained from clams have enhanced therapeutic effects on essential hypertension [14]. These findings demonstrate the significant potential of peptides derived from *Ruditapes philippinarum* in various health applications.

In our previous studies, we found that RPPs possess strong ACE-inhibitory activity [15]. In this study, we further explored the effects of RPPs on deoxycorticosterone acetate (DOCA)-and-salt-induced secondary hypertension in vivo. We investigated their ability to lower blood pressure and assessed their potential to mitigate damage to target organs associated with hypertension in DOCA–salt hypertensive rats. The findings will offer valuable insights into the development of marine functional foods incorporating RPPs for cardiovascular health.

## 2. Results

### 2.1. RPPs Significantly Reduced Blood Pressure in DOCA–Salt Hypertensive Rats

**Figure 1:**
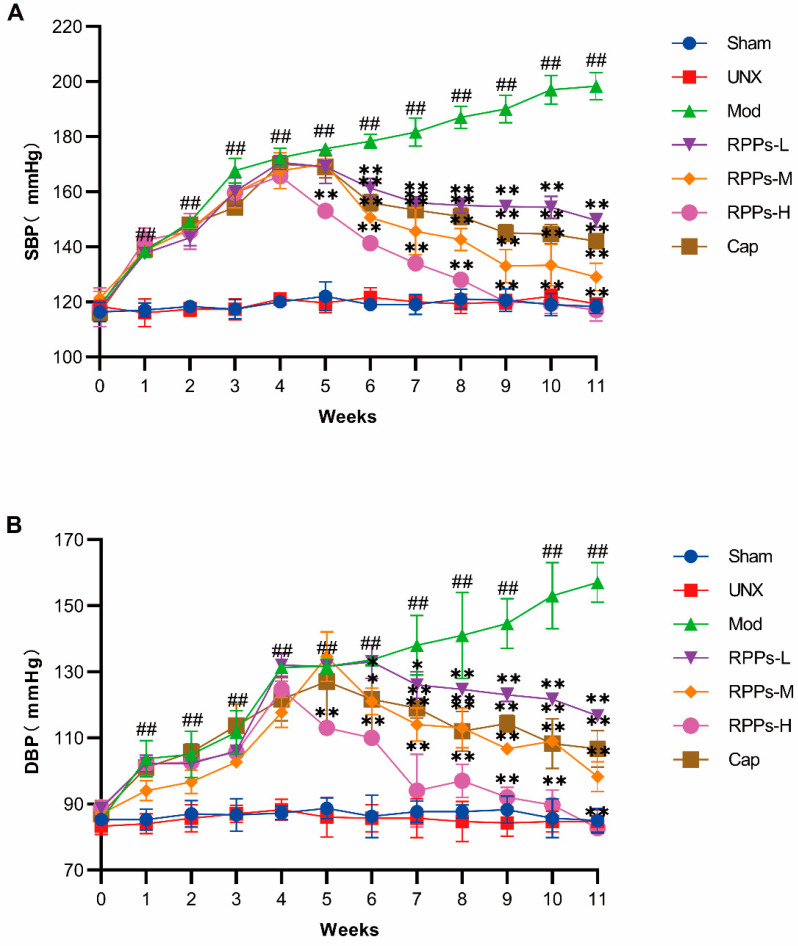
Effect of RPPs on blood pressure in DOCA–salt hypertensive rats. (**A**) Changes in systolic blood pressure over the 11-week experimental period in each rat group. (**B**) Changes in diastolic blood pressure over the 11-week experimental period in each rat group. All data are presented as mean ± SD, ## *p <* 0.01 vs. Sham, * *p <* 0.05, ** *p <* 0.01 vs. Mod.

To evaluate the effect of RPPs on hypertension, we first established a hypertensive rat model using a combination of DOCA and salt. As shown in Figure 1A,B, both systolic and diastolic blood pressure in the Mod group exhibited a continuous increase throughout the modeling period. By the fourth week, the systolic blood pressure in the Mod group reached 172.3 ± 3.5 mmHg, significantly higher than that in the Sham group. Meanwhile, the high-dose RPP intervention resulted in a notable decrease in both systolic and diastolic blood pressure after just one week (*p* < 0.01). From the second week of intervention, all treatment groups exhibited significant reductions in blood pressure, with a dose-dependent response observed across the different RPP dose groups. Notably, the antihypertensive effect of RPPs-H surpassed that of captopril. Particularly, in the RPPs-H group, systolic blood pressure returned to normal levels after five weeks of intervention, while diastolic blood pressure normalized before the end of the experiment. In comparison, captopril did not demonstrate a superior therapeutic effect. Therefore, the high dose of RPPs exhibited a more pronounced hypotensive effect than captopril in the DOCA–salt hypertensive rat model.

### 2.2. RPPs Improved Vascular Remodeling and Attenuated Vascular Fibrosis in DOCA–Salt Hypertensive Rats

**Figure 2:**
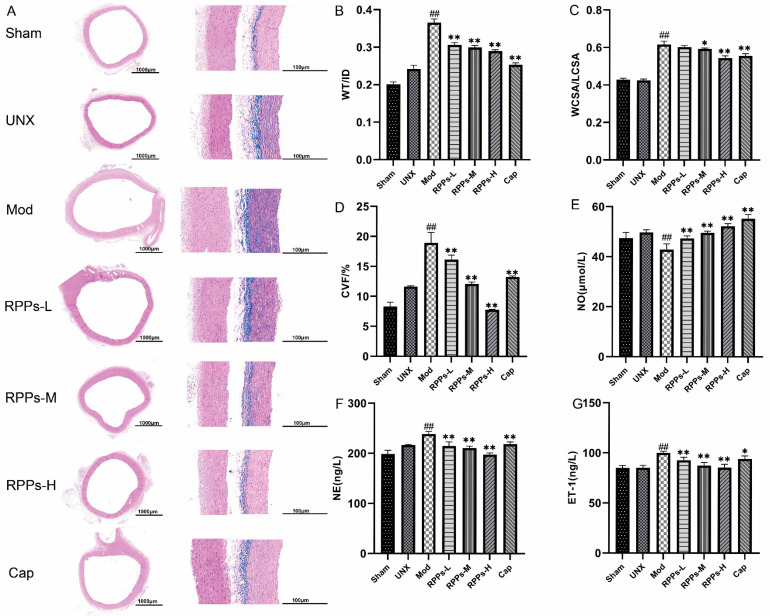
Effects of RPPs on thoracic aortic vascular remodeling in DOCA–salt hypertensive rats. (**A**) Representative micrographs of rat thoracic aorta stained by HE (5×, 40×) and Masson staining (40×); (**B**,**C**) parameters characterizing vascular remodeling, WT/ID and WCSR/LCSR; (**D**) collagen volume fraction; (**E**–**G**) serum NO, NE, and ET-1 concentrations. Data in the graphs are expressed as mean ± SD, ## *p* < 0.01 vs. Sham, * *p* < 0.05, ** *p* < 0.01 vs. Mod.

Long-term hypertension exerts a significant impact on blood vessels, leading to vascular deformation, endothelial damage, and the thickening of the vessel wall. HE staining of aortic sections in the Mod group showed thickening vascular walls, irregular arrangements of endothelial cells, increased density of cell nuclei, and disordered myofibril arrangement, indicating obvious vascular remodeling (Figure 2A). However, treatment with RPPs in each group led to a significant reduction in aortic wall thickness. Masson staining further confirmed the presence of substantial collagen fiber deposition in the thoracic aorta of the Mod group. Meanwhile, the parameters characterizing vascular remodeling (Figure 2B,C), such as WT/ID and WCSR/LCSR, were significantly higher in the Mod group (*p* < 0.01). The results suggested that the blood vessels may have undergone hypertrophic remodeling. Remarkably, the Cap, RPPs-M, and RPPs-H groups all exhibited substantial improvements in these vascular remodeling indexes, with the RPPs-H group showing superior efficacy compared with the Cap group. Furthermore, the RPP treatment led to a notable reduction in collagen fiber deposition in the thoracic aorta, as evidenced by a significant decrease in the CVF (*p* < 0.01) (Figure 2D). Figure 2E–G show that RPPs effectively increased the serum NO concentration while reducing levels of NE and ET-1, which promoted vasodilation, leading to blood pressure reduction. Captopril demonstrated a similar effect, although its blood-pressure-lowering effect was less pronounced. These findings collectively indicated that RPPs exerted a significant improvement effect on vascular remodeling in DOCA–salt hypertensive rats, reducing collagen fiber deposition and attenuating vascular fibrosis.

### 2.3. RPPs Regulated Water–Sodium Balance in DOCA–Salt Hypertensive Rats

**Figure 3:**
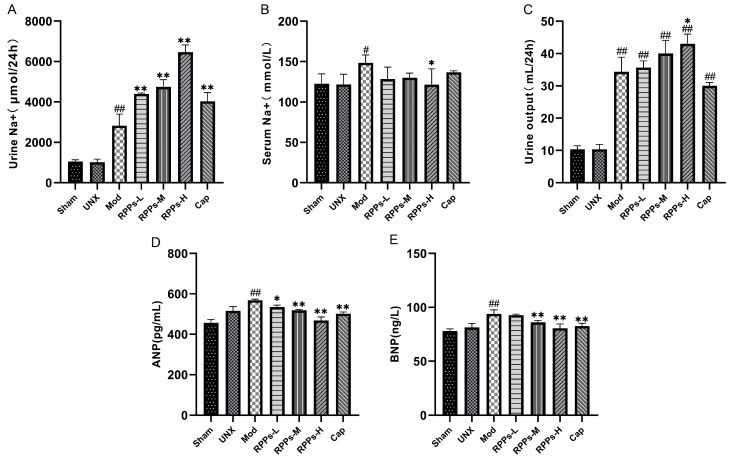
Effect of RPPs on water–sodium balance in DOCA–salt hypertensive rats. (**A**) Urinary Na^+^ concentration; (**B**) serum Na^+^ concentration; (**C**) 24 h urinary output; (**D**,**E**) serum levels of ANP and BNP. All data in the graphs are expressed as mean ± SD, # *p* < 0.05, ## *p* < 0.01 vs. Sham, * *p* < 0.05, ** *p* < 0.01 vs. Mod.

The DOCA–salt hypertension model is commonly used to induce hypertension via DOCA administration along with a high-salt diet, with salt being a significant factor in hypertension formation. To evaluate the effect of RPPs on the water–sodium balance, we measured Na^+^ levels in the urine and blood of rats. As shown in Figure 3A, the 24 h sodium ion excretion was significantly increased in the groups subjected to saltwater consumption, and the RPP intervention groups showed even higher Na^+^ excretion compared with the Mod group (*p* < 0.01). Moreover, the serum Na^+^ level of the Mod group was significantly higher than that of the Sham group (*p* < 0.05). However, the high-dose RPP intervention significantly reduced serum sodium ion levels (*p* < 0.05), while the Cap group did not show a significant reduction (Figure 3B). Figure 3C demonstrates that the 24 h urine volume was significantly higher in all groups on a high-salt diet (*p* < 0.01), with the RPPs-H group exhibiting a significantly higher urine volume compared with the Mod group (*p* < 0.05). ANP and BNP, which are hormones secreted by the heart to regulate the water–sodium balance, were found to be significantly elevated in the Mod group (Figure 3D,E, *p* < 0.01), indicating the body’s compensatory mechanism in maintaining the water–sodium balance. However, the RPPS-M, RPPS-H, and Cap groups showed significant reductions in both ANP and BNP levels (*p* < 0.01), suggesting that the compensatory effect gradually weakened with intervention, following a dose-dependent pattern. These findings illustrated that RPPs present the ability to promote water and sodium excretion, thereby reducing the volume load on the body and ultimately improving hypertension.

### 2.4. RPPs Exerted an Improving Effect on Heart Damage Caused by Hypertension

**Figure 4:**
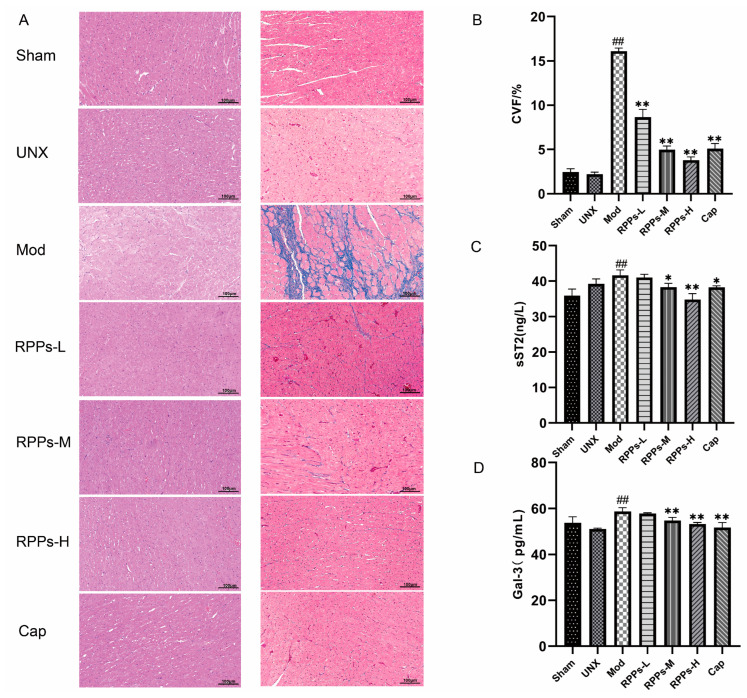
Effects of RPPs on cardiac injury in DOCA–salt hypertensive rats. (**A**) Representative micrographs of HE-stained (40×) and Masson-stained (40×) rat hearts; (**B**) collagen volume fraction; (**C**,**D**) concentrations of serum markers of heart failure (sST2 and Gal-3). All data in the graphs are expressed as mean ± SD, ## *p* < 0.01 vs. Sham, * *p* < 0.05, ** *p* < 0.01 vs. Mod.

Elevated levels of ANP and BNP in the Mod group indicated possible cardiac injury (Figure 3D,E). Histological analysis of heart sections showed the significant enlargement and disorganization of cardiomyocytes in the DOCA–salt hypertensive rats (Figure 4A). Additionally, Masson staining revealed severe fibrosis in the cardiomyocytes, as evidenced by the prominent blue coloration, and the collagen fiber coefficient was significantly increased (Figure 4B). However, the RPP treatment showed a notable amelioration of these pathological changes. The results demonstrated that RPP administration led to a significant reduction in the collagen fiber coefficient (*p* < 0.01) and improved cardiac fibrosis in a dose-dependent manner. Furthermore, serum tests indicated that the levels of Gal-3 and sST2, both markers of heart failure, were significantly elevated in the Mod group (*p* < 0.01). However, in the RPPs-H group, the levels of Gal-3 and sST2 were essentially restored to normal levels (Figure 4C,D). These findings suggested that RPPs exert a significant ameliorative effect on hypertension-induced cardiac injury and have the potential to prevent the development of heart failure.

### 2.5. RPPs Ameliorated Hypertension-Induced Kidney Damage

**Figure 5:**
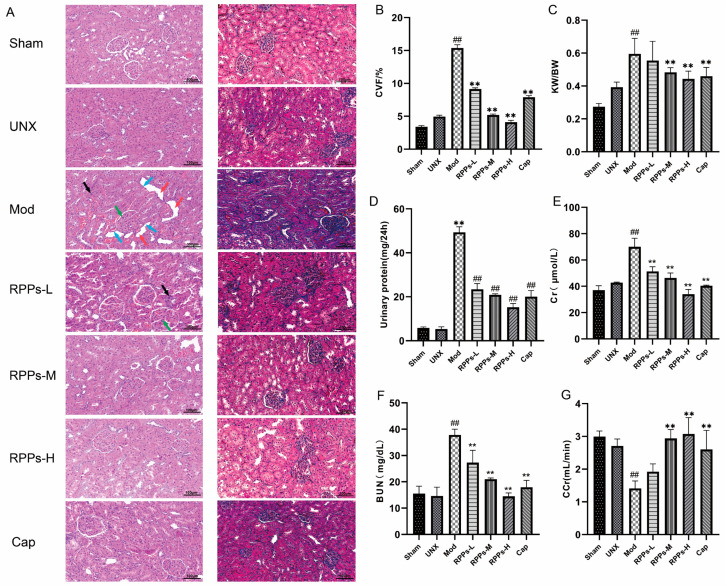
Effect of RPPs on renal function in DOCA–salt hypertensive rats. (**A**) Representative micrographs of HE-stained (40×) and Masson-stained (40×) rat kidney tissues. Inflammatory cell infiltration (black arrow mark), renal glomerular hypertrophy and higher cell numbers (green arrow mark), compensatory dilatation of renal tubules (red arrow mark), flattening and detachment of epithelial cells (blue arrow mark); (**B**) collagen volume fraction; (**C**) renal indices; (**D**) urine protein concentration; (**E**–**G**) serum renal-function-related markers (serum creatinine, BUN, and creatinine clearance rate). All data in the graphs are expressed as mean ± SD. ## *p* < 0.01 vs. Sham, ** *p* < 0.01 vs. Mod.

Persistent hypertension has been shown to contribute to kidney damage [16,17]. HE staining kidney sections from the Mod group revealed inflammatory cell infiltration; renal glomerular hypertrophy and higher cell numbers; the compensatory dilatation of renal tubules; and the flattening and detachment of epithelial cells, indicating renal injury (Figure 5A). However, these pathological changes were alleviated after the RPP intervention. The results of Masson staining also demonstrated severe fibrosis in the Mod group (Figure 5B), which was significantly reduced in all RPP treatment groups (*p* < 0.01), suggesting that RPPs alleviated renal fibrosis. Since the rats in this study underwent left nephrectomy surgery, the UNX group exhibited a compensatory enlargement of the right kidney and an elevated renal index (right kidney weight/body weight) as a normal phenomenon (Figure 5C). Therefore, the Mod group showed a significant increase in the renal index compared with the UNX group, which was significantly ameliorated by medium to high doses of RPPs. Additionally, the Mod group exhibited much higher levels of urinary protein compared with the normal level, and the levels of serum creatinine and urea nitrogen were also significantly increased (Figure 5D–F, *p* < 0.01), resulting in a significant decrease in the creatinine clearance rate (Figure 5G, *p* < 0.01). However, the RPP treatment significantly improved these renal-function-related markers, with the high-dose group showing better efficacy than the Cap group. These findings suggested that RPPs have a significant improvement effect on hypertension-induced renal injury.

### 2.6. RPPs Reduced Oxidative Stress and Inflammatory Responses in DOCA–Salt Hypertensive Rats

**Figure 6:**
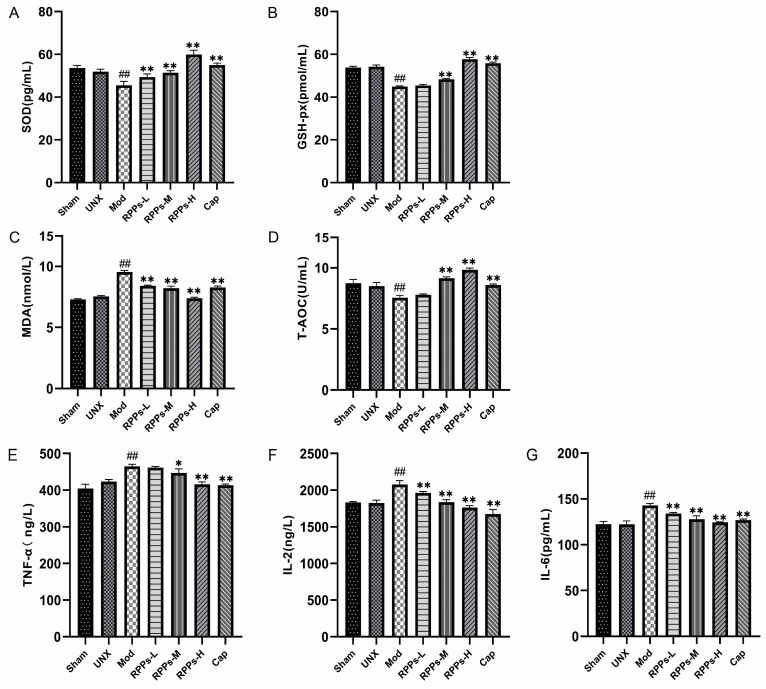
Effect of RPPs on oxidative stress in DOCA–salt hypertensive rats. (**A**–**C**) Levels of SOD, MDA, and GSH-px in kidney tissues; (**D**) T-AOC; (**E**–**G**) serum levels of inflammatory factors TNF-α, IL-2, and IL-6. All data in the graphs are expressed as mean ± SD. ## *p* < 0.01 vs. Sham, * *p* < 0.05 vs. Mod, ** *p* < 0.01 vs. Mod.

To further investigate the protective effects of RPPs on the kidney, we examined the levels of SOD, MDA) GSH-px, and T-AOC in rat kidney tissues. As shown in Figure 6A–D, renal SOD and GSH-px levels were significantly lower (*p* < 0.01) in the Mod group, while the MDA level was significantly higher (*p* < 0.01), and the total antioxidant capacity was also significantly reduced (*p* < 0.01). However, in the captopril and RPP intervention groups, significant increases in SOD levels and decreases in MDA levels were observed (*p* < 0.01). Furthermore, the RPPs-M, RPPs-H, and Cap groups exhibited significantly elevated GSH-px levels and total antioxidant capacity (*p* < 0.01). It has been established that oxidative stress is closely linked to inflammatory responses. The results in Figure 6E–G show that the serum levels of IL-2, IL-6, and TNF-α were significantly elevated in the Mod group (*p* < 0.01). However, all doses of RPPs and captopril significantly reduced the levels of IL-2 and IL-6 (*p* < 0.01). Although low-dose RPPs did not significantly affect TNF-α, medium-dose RPPs managed to reduce its level (*p* < 0.05), and the effect was even more pronounced with high-dose RPPs and captopril (*p* < 0.01). These results indicated that RPPs have the ability to protect the kidney by attenuating renal oxidative stress and suppressing the inflammatory response.

### 2.7. RPPs Had a Positive Regulatory Effect on the Gut Microbiota of DOCA–Salt Hypertensive Rats

**Figure 7:**
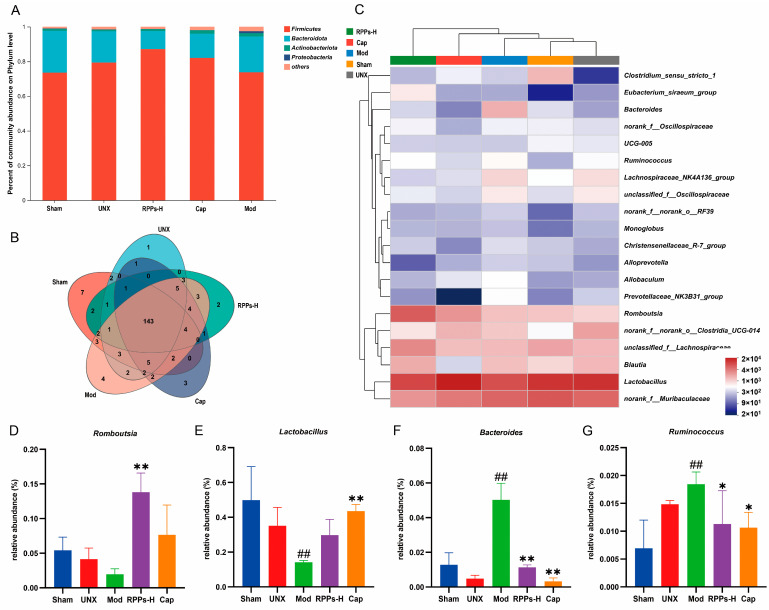
Effect of RPPs on gut microbial composition and diversity in DOCA–salt hypertensive rats. (**A**) Proportion of the top 5 species composition at the phylum level; (**B**) Venn plot at the genus level; (**C**) heat map analysis of the community at the genus level. (**D**–**G**) Amount of *Romboutsia*, *Lactobacillus*, *Bacteroides*, and *Ruminococcus* in the fecal microbiota. All data in the graphs are expressed as mean ± SD. ## *p* < 0.01 vs. Sham, * *p* < 0.05 vs. Mod, ** *p* < 0.01 vs. Mod.

We analyzed the gut microbiota in rat feces to explore the relationship between RPPs and gut microbiota in improving DOCA–salt hypertension. When comparing the relative amount of the top five most abundant phyla in the five groups (Figure 7A), it was observed that the Mod group had a relatively high amount of Proteobacteria compared with the Sham group, whereas their amount was significantly lower in the RPPs-H group. Further analysis at the genus level revealed four endemic genera in the Mod group, namely, *Sutterella*, *Mycoplasma*, *Morganella*, and *Anaerosalibacter* (Figure 7B). Among these, *Sutterella* and *Mycoplasma* are associated with inflammation. Additionally, a genus-level community heat map analysis (Figure 7C–G) showed that the RPP treatment led to an increase in the amount of beneficial bacteria, specifically, *Romboutsia* and *Lactobacillus*, while reducing the amount of *Bacteroides* and *Ruminococcus*, which are both associated with pathogenic activity. These results suggest that RPPs have the potential to regulate the balance and stability of the gut microbiota by promoting the growth of beneficial bacteria and suppressing the growth of harmful bacteria.

## 3. Discussion

Hypertension poses a significant threat to human health, with a high incidence rate worldwide. The current scientific consensus attributes hypertension to an interplay between genetic and environmental factors. Given its complex etiology, patients often require customized antihypertensive treatments, including the use of different drugs or combination therapies, to effectively manage blood pressure [18]. In our previous study, we found the robust ACE-inhibitory activity of RPPs [15]. To further investigate whether RPPs possess other antihypertensive mechanisms, we employed DOCA–salt hypertensive rats, which simulate salt–corticosteroid hypertension accompanied by renal impairment. This model is characterized by sodium retention, oxidative stress, elevated inflammatory markers, and fibrosis in target organs [19]. In the present study, RPPs exerted significant antihypertensive effects on DOCA–salt hypertensive rats, surpassing the impact of the ACE inhibitor captopril. These findings suggest that RPPs may hold promise in ameliorating hypertension arising from diverse underlying factors. This underscores their potential application as functional food ingredients or even therapeutic agents.

In this study, DOCA–salt hypertensive rats showed a significant upward trend in SBP and DBP. However, the RPP treatment effectively reduced them, surpassing the efficacy of captopril. Remarkably, the high dose of RPPs did not further lower blood pressure beyond the normal range, indicating its potential as a safer alternative to traditional antihypertensive medications. Long-term hypertension can lead to pathological changes in blood vessels. Vascular remodeling, a pathological alteration in the vascular wall structure, can lead to compromised vascular function and organ damage [20]. A previous study suggested that during the treatment of Ang II-induced hypertension in mice, lowering blood pressure was accompanied by a significant decrease in vascular and cardiac tissue remodeling [21]. In the Mod group, a pathological examination of aortic sections showed significant vascular wall thickening with a substantial deposition of collagen fibers. RPPs significantly attenuated these pathological changes. This indicates that RPPs may reduce blood pressure by relaxing blood vessels to prevent vascular remodeling, thereby reducing cardiac load and preventing heart damage. Serum assays further exhibited a decline in NO concentration, concomitant with an increase in NE and ET-1 levels, indicating the occurrence of vascular remodeling in DOCA–salt hypertensive rats. ET-1 is a potent vasoconstrictor peptide and a crucial regulator of blood flow. A reduction in ET-1 levels and an increase in NO levels are commonly associated with vasodilation [22]. This further supports the idea that RPPs could lower blood pressure by relaxing blood vessels. Meanwhile, previous studies have reported that captopril can effectively dilate blood vessels and lower blood pressure by increasing NO levels [23]. In our study, RPPs exhibited superior efficacy compared with captopril in enhancing endothelial functions and promoting vasodilation. To better evaluate vascular function, we will compare the systolic function of arteries to further illustrate the role of RPPs in vasodilation.

Hypertension development in DOCA–salt hypertensive rats involves complex mechanisms, such as the dysregulation of the renal Na^+^-handling capacity and water and sodium retention [24]. In our study, the model rats were exposed to high saline intake, leading to a notable increase in Na^+^ levels in vivo. To maintain a relatively stable water–sodium balance, the rats had to rely on increased vascular pressure to promote Na^+^ excretion. However, the sustained elevation of blood pressure could result in renal injury, further impacting water–sodium excretion and creating a vicious cycle [25]. Similar to the findings of Boesen EI et al. [26], our study demonstrated that urine volume, urine Na^+^, and serum Na^+^ were significantly higher in the Mod group compared with the Sham group because of heavy salt water consumption. However, the urine volume and Na^+^ of the Mod group were lower than the RPPs-H group. This suggests that the intervention with RPPs promoted water and Na^+^ excretion, effectively preventing the occurrence of water and sodium retention. ANP and BNP are diuretic and natriuretic peptide hormones secreted by the heart [27]. A long-term high-salt diet in DOCA–salt rats led to disruptions in water–sodium metabolism, leading to the increased expression of ANP and BNP. However, the effects of these hormones are attenuated during heart failure [28], and some studies have suggested a positive correlation between ANP and BNP levels with blood pressure [29]. Remarkably, the RPP intervention significantly reduced the levels of ANP and BNP, and its efficacy surpassed that of captopril. The results suggest that RPPs play a vital role in regulating the water–sodium balance, which could be one of the reasons underlying its superior antihypertensive effect compared with captopril.

Elevated levels of ANP and BNP are also known to be compensatory homeostatic responses to myocardial overload [30], indicating that hypertension can lead to cardiac damage. The pathological sections showed abnormal hypertrophy of the myocardial cells and severe fibrosis in the Mod group. However, RPPs had an obvious ameliorative effect on cardiac damage. Reducing BNP is considered to have high prognostic value in clinical practice, as its levels decrease with the treatment of heart failure [31]. In the study conducted by Huang, it was shown that potato hydrolysate peptides had the ability to ameliorate cardiac hypertrophy and fibrosis in SHR rats, with a reduction in BNP expression levels [32]. These findings were consistent with our research, where the intervention using RPPs led to a notable decrease in BNP levels. Additionally, sST2 and Gal-3 are fibrosis markers that are also prognostic indicators of heart failure [33]. The RPP treatment caused a reduction in both sST2 and Gal-3 levels, which, in combination with the decrease in ANP and BNP levels, demonstrated that the RPPs effectively prevented the development of heart failure.

The kidney is a critical target organ of hypertension, and proteinuria is strongly associated with arterial hypertension in patients with chronic kidney disease (CKD) [34]. Prolonged hypertension can lead to renal insufficiency, characterized by glomerulosclerosis; interstitial fibrosis; proteinuria; and, ultimately, a decline in the glomerular filtration rate [35]. In our study, the RPP treatment led to a significant improvement in glomerular lesions and interstitial fibrosis caused by hypertension. The decrease in urinary protein excretion and the recovery of CCr are important indicators of renal injury improvement [36]. The significant elevation of serum BUN, creatinine, and urinary protein levels in the Mod group indicated severe renal injury, which was essentially restored to normal levels with the RPP intervention, thereby providing kidney protection. Similarly, Guan et al. reported that the biologically active peptide apelin could normalize serum creatinine and urea nitrogen levels while also normalizing urinary NGAL and Kim-1 levels, thereby exerting a renoprotective effect [37].

Oxidative stress occurs because of the excessive generation of highly reactive oxygen species (ROS) and nitrogen species (RNS), resulting in cellular damage. In patients with CKD, elevated oxidative stress is associated with various complications, including hypertension, atherosclerosis, inflammation, and anemia [38]. The results showed that the levels of SOD and GSH-px in the kidney tissue of the Mod group rats were significantly reduced, while the levels of MDA were significantly increased, resulting in a significant decrease in total antioxidant capacity. Li Zeng et al.’s study suggests that almond supplementation can prevent the development of salt-induced hypertension by reducing oxidative stress [39]. Similar to this, the high-dose RPP intervention led to a significant increase in the antioxidant capacity of the rats, effectively attenuating oxidative stress and exhibiting a more potent therapeutic effect than captopril in several indices. Inflammation accelerates the development of salt-sensitive hypertension and leads to malignant conditions with end-organ damage [40]. Previous research has demonstrated that inhibiting TNF-α could hold significant therapeutic implications for treating hypertension and its associated end-organ damage [41]. The high doses of RPPs significantly decreased serum TNF-α levels, as well as the levels of other inflammatory factors like IL-2 and IL-6. These findings suggest that RPPs have the ability to alleviate the damage caused by hypertension to target organs, particularly the kidneys, by improving antioxidant capacity and reducing the inflammatory response in DOCA–salt hypertensive rats.

In addition, a high-salt diet can influence blood pressure through its impact on gut microbiota [42]. Interestingly, the Mod group showed a significant increase in Proteobacteria, which include various known pathogenic bacteria such as *E. coli*, *Salmonella*, and *Shigella*. They commonly cause metabolic disorders and trigger inflammatory responses [43]. However, the high-dose RPPs reduced the abundance of Proteobacteria. *Lactobacillus* is known to play a crucial role in regulating the gut microbiota. Previous studies have shown that a high-salt diet can reduce *Lactobacillus* abundance in mice, and salt-sensitive hypertension can be improved by supplementing with *L. murinus* [44]. Our study also revealed that the RPP intervention upregulated the amount of Lactobacillus, contributing to a reduction in blood pressure. Another significant finding was the upregulation of the amount of *Romboutsia* following the RPP intervention. *Romboutsia* is involved in the production of short-chain fatty acids and has been identified as a potent predictor of hypertension. Reduced *Romboutsia* presence has been observed in patients with hypertension [45]. Furthermore, our study demonstrated that the RPP intervention led to a significant downregulation of the amount of *Ruminococcus*, as well as the conditionally pathogenic bacterium Bacteroides. *Ruminococcus* is known to produce inflammatory polysaccharides that disrupt intestinal barrier function [46], and its reduction has been associated with improved hypertension [47]. These results suggest that RPPs could contribute to the maintenance of gut microbiota stability.

This study has potential limitations. Firstly, further research is needed to identify the peptide sequences of RPPs for activity verification to determine the actual effectiveness of the screening sequence and the impact of synergistic effects. Secondly, additional measurements of ACE-inhibitory activity in blood vessels and organs are necessary to further elucidate the antihypertensive effect of RPPs.

## 4. Materials and Methods

### 4.1. Materials

Fresh clams (*Ruditapes philippinarum*) were collected from the reputable Hongdao Aquatic Market in Qingdao, China. *Ruditapes philippinarum* peptides were obtained through a well-established process involving hydrolysis with complex proteases, separation, and purification, following methods described in previous studies. The molecular weight distribution of the RPPs, as determined with high-performance liquid chromatography (HPLC), was 94.29% for peptides with Mw ≤ 1000 Da, 5.41% for 1000 < Mw ≤ 3000, and 0.16% for Mw > 3000. RPPs showed high ACE-inhibitory activity with an IC_50_ value of 0.67 mg/mL [15,48]. Deoxycorticosterone (DOCA) was sourced from Shanghai McLean Biochemical Technology Co. Ltd. (Shanghai, China), while captopril was acquired from Shandong Xinhua Pharmaceutical Co., Ltd. (Zibo, China). The complex protease utilized in the peptide preparation was provided by Shanghai Yuanye Biotechnology Co., Ltd. (Shanghai, China). ELISA kits for blood sodium and urine sodium measurements were provided by the Nanjing Jiancheng Bioengineering Institute (Nanjing, China), and other kits were purchased from Jiangsu Jingmei Biotechnology Co., Ltd. (Shenzhen, China).

### 4.2. Animals and Experimental Design

All experimental procedures involving animals were authorized by the Animal Care and Use Ethics Committee of Qingdao University and strictly abided by the ethical welfare requirements for animals of Qingdao University (Animal Ethics Certificate NO. QDU-AEC-2023344). Healthy male Sprague Dawley (SD) rats weighing between 180 and 220 g were provided by SPF (Beijing) Biotechnology Co. Ltd. All rats were housed in the laboratory animal room with 22 ± 3 °C, 65 ± 5% humidity, and 12 h light/dark cycle and provided with standard laboratory food and water. One week after adaptive feeding, rats underwent left kidney removal surgery (Sham group underwent the same procedures except for kidney removal). After surgery, they were injected with penicillin for three consecutive days to prevent infection. After a week of recovery, the rats were randomly divided into 7 groups (*n* = 8): Sham group (underwent sham surgery); unilateral nephrectomy group (UNX); model group (Mod); low-dose RPP group (RPPs-L); medium-dose RPP group (RPPs-M); high-dose RPP group (RPPs-H); captopril group (Cap). The Sham and UNX groups drank tap water, while the other five groups drank water containing 0.9% NaCl and 0.2% KCl; additionally, each week, 35 mg/kg of DOCA was injected subcutaneously to induce the model establishment [49,50].

After four weeks of modeling, the intervention began. The three RPP groups received daily gastric gavage of RPPs (RPPs-L group: 100 mg/kg, RPPs-M group: 200 mg/kg, and RPPs-H group: 400 mg/kg), while the Cap group received daily gastric gavage of 50 mg/kg captopril (the dosage range of RPPs used is based on previous laboratory research) [48]. The intervention continued for 7 weeks until the end of the experiment. Throughout the experimental period, blood pressure measurements were taken from all rats using the tail-cuff method once a week, with three readings per rat and taking the average value [51]. In addition, the blood pressure was adaptively measured daily for one week before the formal experiment. Metabolic cages were used to collect 24 h urine. At the end of the experiment, rats were anesthetized. Blood samples were collected and centrifuged to obtain serum. Organs were collected and weighed for subsequent analysis and testing.

### 4.3. Pathological Sections and Histopathological Analysis

Paraffin sections of the tissues were fixed using established methods from the literature [52]. Pathology staining was employed to analyze the tissue pathology. The cross-sectional areas of the thoracic aortic wall (WCSR), lumen (LCSR), internal diameter (ID), and external diameter (WD) were measured and calculated using Case Viewer 2.4 and the Image J-win64 software. The collagen volume fraction (CVF) of the kidney, myocardium, and thoracic aorta was quantified and calculated using ImageJ.

### 4.4. Biochemical Analysis of Serum and Kidney Tissue

Serum levels of urea nitrogen (BUN), creatinine (Cr), Na^+^, urinary protein, and urinary Na^+^ were determined using an automatic biochemical analyzer. Additionally, serum levels of nitric oxide (NO), norepinephrine (NE), endothelin-1 (ET-1), galectin-3 (Gal-3), soluble suppression of tumorigenicity-2 (sST2), tumor necrosis factor-alpha (TNF-α), interleukin-2 (IL-2), interleukin-6 (IL-6), atrial natriuretic peptide (ANP), and brain natriuretic peptide (BNP) were measured with their corresponding ELISA kits (Jiangsu Jingmei Biological Technology Co., Ltd., Yancheng, China). For renal tissue, superoxide dismutase (SOD), malondialdehyde (MDA), glutathione peroxidase (GSH-px), and total antioxidant capacity (T-AOC) were assessed using ELISA kits.

### 4.5. Analysis of Gut Microbial Diversity

Rat feces were collected one day before the end of the experiment and immediately stored at −80 °C. Total DNA extraction of the microbial community was carried out using the E.Z.N.A.^®^ soil DNA kit (Omega Bio-tek, Norcross, GA, USA). Sequencing was performed using the Illumina Miseq PE300/NovaSeq PE250 platform. The raw sequences obtained were subjected to quality Sham and splicing. OTU clustering was performed based on 97% similarity, and chimeras were excluded. Each sequence was annotated with species classification and compared with the Silva 16S rRNA database (v138) at a comparison threshold of 70%. Sequencing results were analyzed using the Majorbio Cloud platform (https://cloud.majorbio.com/, accessed on 20 August 2023).

### 4.6. Statistical Data Analysis

All data were recorded as mean ± SD. After performing the normality test, one-way ANOVA was utilized to analyze significant differences, followed by Tukey’s test. Statistical significance was considered at *p* < 0.05, and high significance at *p* < 0.01. SPSS 19.0 was used for all data analysis.

## 5. Conclusions

Our study demonstrated that RPPs exerted a significant hypotensive effect on DOCA–salt hypertensive rats. This effect is achieved by promoting water and sodium excretion, upregulating NO levels, and reducing ET-1 levels. Meanwhile, RPPs can effectively reduce vascular remodeling and prevent the occurrence of heart failure. Furthermore, RPPs can significantly alleviate kidney damage caused by hypertension, alleviate oxidative stress in the kidneys, and inhibit the elevation of inflammation levels. RPPs also showed modulatory effects on gut microbiota. Based on the findings of our study, we conclude that RPPs have an obvious improvement effect on essential hypertension and secondary hypertension caused by DOCA–salt and can effectively alleviate organ damage caused by hypertension.

## Data Availability

All data that support the findings of this study are available from the corresponding authors upon reasonable request.
